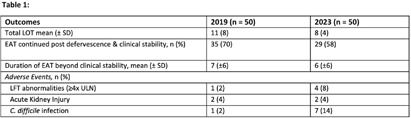# Evaluation of Empiric Antibacterial Treatment and Subsequent De-escalation for Febrile Neutropenia

**DOI:** 10.1017/ash.2024.256

**Published:** 2024-09-16

**Authors:** Eunice Kim, Christopher McCoy, Ryan Chapin

**Affiliations:** Beth Israel Deaconess Medical Center

## Abstract

**Background:** Febrile neutropenia (FN) is the most common complication of chemotherapy-induced neutropenia that affects over 80% of patients with hematologic malignancies. National guidance and randomized controlled trial data demonstrate empiric antimicrobial therapy (EAT) can be discontinued after 72 hours of apyrexia and clinical recovery regardless of absolute neutrophil count (ANC). A 2019 internal study identified opportunity for improvement for targeted de-escalation. We aimed to reevaluate duration of EAT in patients with FN without a documented source of infection. **Methods:** A pharmacovigilance platform identified 110 patients from January to September 2023 without identified source of infection. Data collection was performed via manual chart review. Historic patient data from our 2019 cohort (n=50) was available in our research repository. The primary outcome was the duration of EAT in patients with at least 72 hours of apyrexia and clinical recovery, defined as normalization of vital signs. Secondary outcomes included adverse events associated with EAT, and initiation of intravenous vancomycin. **Results:** Baseline characteristics for 2023 were similar to historic, median age was 67.5 years, 56% were male, and median ANC at fever onset was 150 cells/μL. EAT was continued in 29 patients (58%) despite defervescence and stabilization versus 35 (70%) in 2019 (figure 1). Average duration (LOT) of EAT beyond clinical stabilization was 6 versus 7 days. Adverse effects due to EAT occurred in 13 patients in 2023 (26%) versus 4 (8%); of which, C. difficile infection (CDI) was the greatest contributor. Vancomycin was initiated in 31 patients (62%), 22 having no identifiable indication. **Conclusions:** Rates of EAT de-escalation for neutropenic patients after 72 hours of apyrexia and clinical stability improved by 12% as compared to 2019. Mean days of overall EAT was 3 days less in 2023. With a notable increase in CDI rates in 2023, dedicated time for antimicrobial stewardship review, clinician education and guideline driven alerts for review will be explored to help further improve practice.

**Figure:**
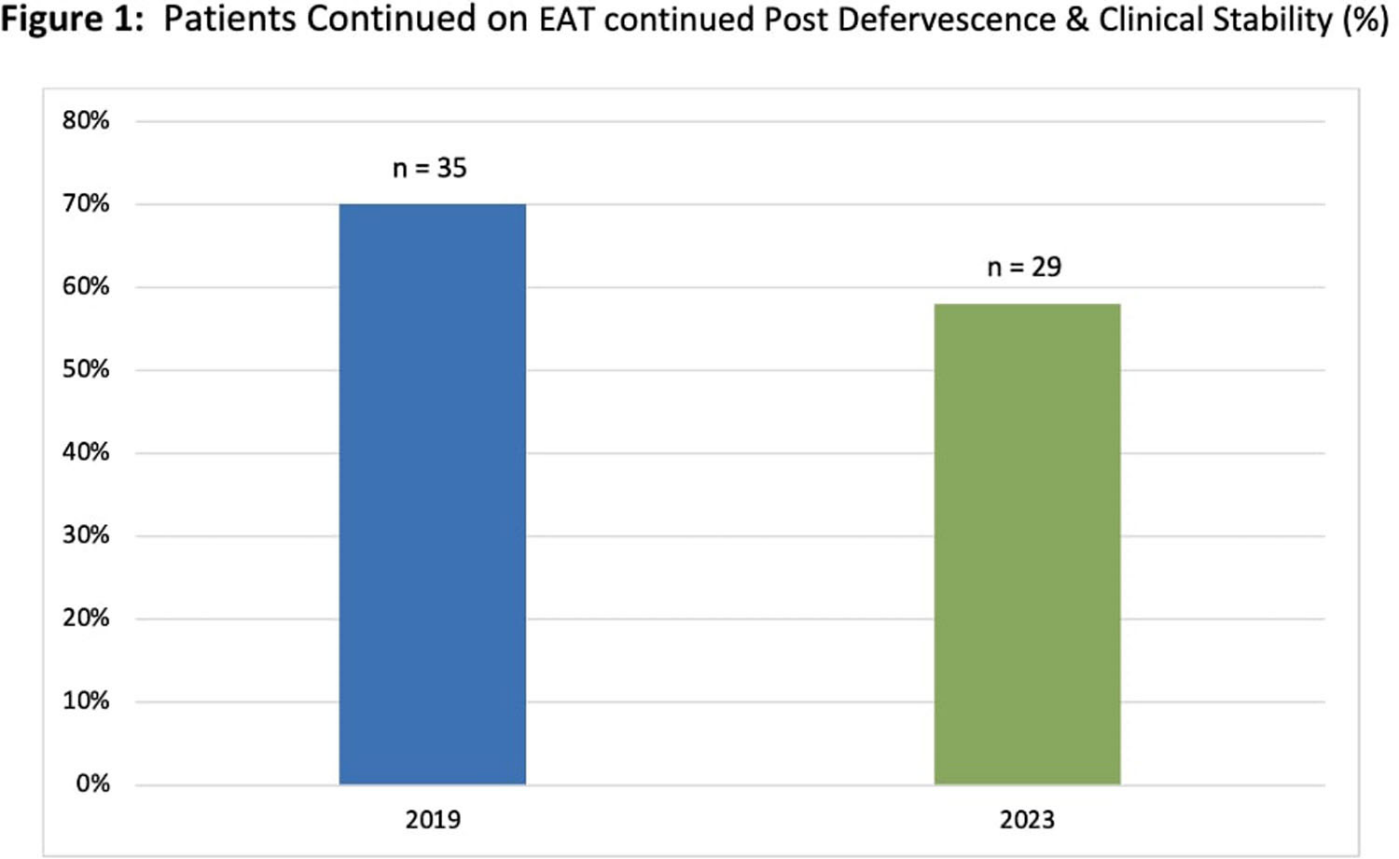

**Figure:**